# Aerogel Technology for Thermal Insulation of Cryogenic Tanks—Numerical Analysis for Comparison with Traditional Insulating Materials

**DOI:** 10.3390/gels9040307

**Published:** 2023-04-06

**Authors:** Matteo Sambucci, Federico Savoni, Marco Valente

**Affiliations:** 1 Department of Chemical Engineering, Materials, Environment, Sapienza University of Rome, 00184 Rome, Italy; federico.savoni@gmail.com (F.S.); marco.valente@uniroma1.it (M.V.); 2 INSTM Reference Laboratory for Engineering of Surface Treatments, UdR Rome, Sapienza University of Rome, 00184 Rome, Italy

**Keywords:** LNG, fiber-reinforced aerogel blankets, cryogenic tank, thermal insulation, boil-off rate, finite element method

## Abstract

The traditional choice of insulation material for liquefied natural gas (LNG) transportation with cryogenic tankers is the back-filled perlite-based system. However, aiming to further cut down the insulation cost, spare additional arrangement space, and provide safety in installation and maintenance, the requirement of looking for alternative materials still exists. Fiber-reinforced aerogel blankets (FRABs) could represent good candidates in designing insulation layers for LNG cryogenic storage because of their ability to ensure adequate thermal performance without the need to create deep vacuum conditions in the annular space of the tank. In this work, a finite element method (FEM) model was developed to study the thermal insulation performance of a commercial FRAB (Cryogel ^®^ Z) for application in cryogenic storage/transport LNG tanks, comparing it with the performance of traditional perlite-based systems. Within the reliability limits of the computational model, the analysis proved that FRAB insulation technology gave encouraging results and might be potentially scalable for transporting cryogenic liquid. In addition to demonstrating superior performance in terms of thermal insulating efficiency and boil-off rate over the perlite-based system, as far as a perspective of cost savings and space gain, FRAB technology allows for higher levels of insulation without vacuum and with lower thickness of the outer shell, which is therefore beneficial for storing more material and lightening the weight of the LNG transportation semitrailer.

## 1. Introduction

In the current era, the growing interest in liquefied natural gas (LNG) is related to the general increase in the demand for natural gas. LNG is a relatively cheap fuel with high ecological value [1]. As claimed by Gustafsson and Svensson [2], the implementation of LNG in place of fossil-based diesel can potentially establish reductions in greenhouse gas (GHG) emissions of up to 15% from vehicles [2]. Based on the above-mentioned evidence, LNG is expected to play a big role in the future energy policies of various countries in the European Union (EU). According to the European Union’s Green Book, there is a need to replace 20% of conventional fuel consumption with alternative fuels within this decade [3], and LNG may represent one of the primary candidates for this purpose.

In recent years, natural gas has been increasingly stored and transported in liquid form because LNG has approximately one-six hundredth of its volume when in the gaseous state. Its reduction in volume will increase the energy density stored in the fuel and make LNG more reliable and practical to store and transport to long distances where the pipeline networks are infeasible [4]. In addition, liquefaction involves treatments that make LNG free of impurities and moisture [1,5]. One of the main concerns in the management of LNG storage tanks, which should be kept at very low temperatures (from −150 °C to −200 °C for the time necessary for delivery), is the vaporization of the liquid caused by heat absorption from the surrounding environment. This issue is essential for the reduction of financial loss and for the prevention of possible safety risks, such as damage in tanks [5]. The conventional storage LNG systems are designed as double-walled tanks with deep-vacuum annular space, fully or partially filled by insulation material. The internal vessel and external jackets are usually made of austenitic stainless steel, which is selected to preserve mechanical ductility at very low temperatures [1]. Thermal-insulating materials for cryogenic applications can be divided into three groups: foams, bulk-fill, and layered. Foam-based insulators, including polyurethane or polystyrene foam, can be implemented in “no-vacuum” conditions, providing thermal conductivity value in the range of 35–55 mW/m × K. However, their use in cryogenic mobile tanks is strongly discouraged because of the marked tendency for low-temperature structural degradation and flammability [1]. Bulk-fill and layered systems represent the major solutions for designing the heat insulation part of the mobile tank. In terms of thermal resistivity, multi-layer insulation (MLI) is the configuration that establishes the best performance, ensuring thermal conductivity values lower than 0.03 mW/m × K [1,6]. The effectiveness of MLI strictly depends on the vacuum level. It requires a vacuum level below 10^−3^ Pa to be fully effective [6]. The vacuum level often degrades to the point where the potential performance of MLI is lost, causing a dangerous increment in pressure into the inner tank. Additionally, the stages of evacuation, heating, and vacuum retention are expensive and time-consuming, as is the setting of MLI insulation inside the inner vessel [7]. The bulk-fill systems would seem to provide the best combination of thermal performance, low cost, safety, and minimal maintenance. The most employed microporous material used for back-filling of the cryogenic tank is expanded perlite, with bulk density from 50 to 300 kg/m^3^ and thermal conductivity coefficient 1–2 mW/m × K under not excessively deep vacuum conditions (around 0.1 Pa) [6]. Although inexpensive and user-friendly to utilize as bulk evacuated insulation, perlite tends to compact and settle under the inner vessel during thermal cycling. The compaction increases heat loss to the inner vessel and can damage internal piping and supports. In addition, water adsorption is also a noteworthy problem for perlite-insulated cryogenic tanks. Perlite insulation adsorbs water to the point that it is impossible to achieve an acceptable vacuum level. The results of these drawbacks are increased evaporation rate with age, costly replacement of bulk insulation, and possible repair of internal components [7]. In this scenario, the research is facing new cryogenic insulating solutions to overcome the issues discussed above.

Aerogels are highly porous open-cell solid materials (~90% open porosity) prepared by a supercritical drying process. The extremely low thermal conductivity (0.013 W/m × K) makes aerogels attractive thermal super-insulators mainly by minimizing heat conduction through their low density (80–200 kg/m^3^) and tortuous solid nanostructure (mesopore diameters between 4 and 20 nm) [8,9]. However, this is obtained at the expense of physical and mechanical properties. The low compressive strength and high susceptibility to fracture make them difficult to handle, and the damage can negatively affect the thermal performance. Additionally, aerogels are also prone to settling over time, especially when exposed to vibration or thermal cycling [1,8]. To improve the durability and structural characteristics of aerogels for heat insulation applications, fiber-reinforced aerogel blankets (FRABs) have been developed. Typically, a FRAB is composed of aerogel particles covering a network of reinforcing fibers (polymer or ceramic) acting as a supporting skeleton. The random porosity is due to the void spaces between the covered fibers [10]. The implementation of fibers has undoubted advantages for strengthening purposes while maintaining extremely low thermal conductivity (ranging between 0.017 and 0.04 W/m × K), almost comparable to that of “pure” aerogel [8]. Due to their unique properties, these materials have recently been used for various ground and aerospace applications, such as [11,12]: (a) thermal insulators in building of industrial pipelines, (b) fire retardant systems, (c) noise insulation panels for acoustic control in situations like offices or recording studios, (d) propellant spray foams onto refrigerator walls instead of pullulating chlorofluorocarbon (CFC) fluids, (e) spacecraft thermal control systems, and (f) cryogenic propulsion tanks.

Commercially, for the last 20 years, Aspen Aerogels Inc. (Northborough, MA, USA) has manufactured several different types and formulations of FRABs for industry use. In the special framework of cryogenic applications, Cryogel ^®^ Z is a fiber-reinforced aerogel-based insulation blanket in polyethylene terephthalate (PET) or polyester and glass fiber continuous filaments, designed to provide maximum thermal protection at low thickness and with a very low specific weight. It is a product developed for use at low temperatures, with a working range between −200 °C to +90 °C. The manufacturing process, patented by Aspen Aerogels Inc., consists of infiltrating a sol mixture into a fibrous web in an automated roll-to-roll device. The sol is catalyzed to convert to a gel within the interstitial spaces of the web before subsequent processing steps. The gelation step is followed by an aging stage in a solution that improves strength and critical performance properties, such as thermal conductivity and hydrophobicity. The final step in the process is to dry the wet gel under supercritical carbon dioxide (CO_2_) conditions. The use of supercritical CO_2_ for the removal of gel solvent from the thick gel blanket composites for large-scale processing has proven both safe and cheap [13]. Some researchers successfully explored the performance of Cryogel ^®^ Z for cryogenic industrial applications, highlighting some advantages over conventional insulating materials. These benefits include a decreased insulation thickness requirement in comparison to conventional insulators (perlite and glass/polyurethane foams), a simplified insulation system design (i.e., no contraction joints), and fast and user-friendly installation, even during operating conditions [13]. In addition, Cryogel ^®^ Z offers thermal insulation characteristics close to the “target” requirement perlite-based bulk-filled system at ambient pressure [14]. The implementation of FRAB technology in LNG cryogenic tanks would therefore bring potential added value:

- To store larger quantities of LNG for the same volume of the tank.
- To reduce considerably the total weight of the tank per equal volume transported, with a clear decrease in transport costs and fuel consumption.
- To realize adequate thermal insulation conditions without vacuum with positive effects in terms of safety, cost savings, and simplified design.

**Figure 1:**
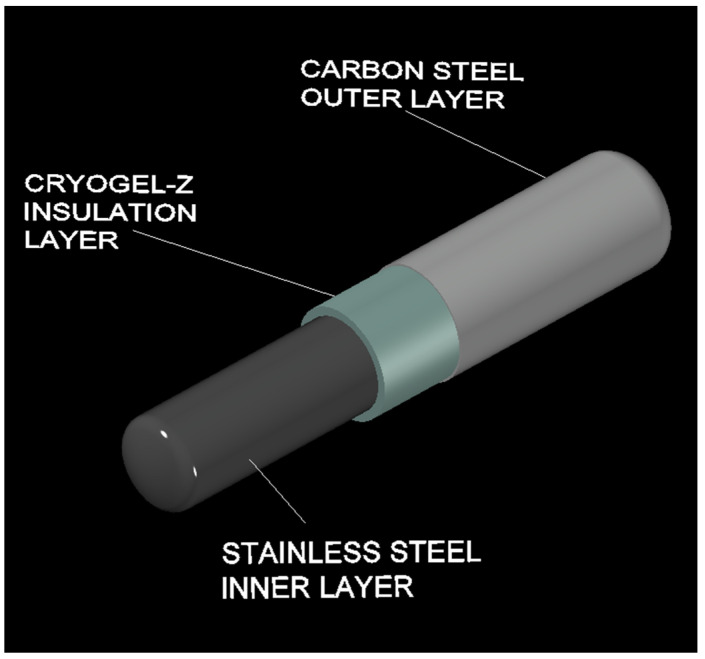
3D render of a cryogenic tank for LNG implemented with a FRAB insulation layer.

In light of the technological advancement that aerogel technology can confer in the LNG cryogenic transportation sector, the purpose of this study deals with an investigation, by finite element method (FEM)-based numerical analysis, of the viability of using FRAB in replacing the insulating configurations currently practiced in the cryogenic tanks (i.e., perlite + vacuum). A 3D render of the cryogenic tanker design for LNG implemented with Cryogel ^®^ Z is shown in Figure 1. Numerical studies were performed using the COMSOL Multiphysics cross-platform software. Specifically, a computational model of a cryogenic tank was built to perform a systematic comparative analysis between FRAB and perlite-based systems, in terms of boil-off rate (*BOR*) and thermo-insulation efficiency (ε), investigating the influence of the insulator thickness (*h*) and storage/transportation time (*t_st_*) under real conditions currently adopted in the design of cryogenic road tankers. To the best of the authors’ knowledge, only a few publications report studies on the use of a FRAB system for LNG cryogenic storage. Therefore, the preliminary results of this work are framed in the implementation of new technologies and materials in the cryogenic transport sector aimed at overcoming critical issues in terms of costs, safety, sustainability, and efficiency that are encountered with traditional insulating systems.

## 2. Results and Discussion

### 2.1. Thermo-Insulation Efficiency (ε) as a Function of Thickness (h)

**Figure 2:**
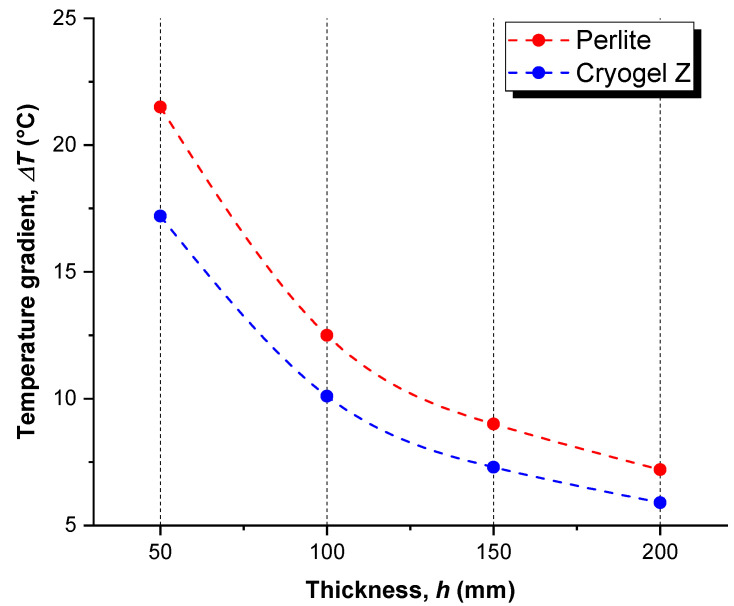
Computed temperature gradient (Δ*T*) within the LNG domain after 96 h storage as a function of insulator thickness (*h*).

Figure 2 plots the influence *h* on the temperature gradient (Δ*T*) developed within the LNG domain after 96 h storage, which is an estimate of the time it takes for a cryogenic truck to cover 1000 km distance. The results provide a first quantitative assessment of the ability of the insulating systems under examination to avoid heat leakage and maintain an adequate temperature of LNG throughout its transportation [15].

Regardless of the type of insulating system, it was predictable that, as *h* increases, Δ*T* tends to progressively decrease because of a better-established heat insulation effect. Thermal transmission in a certain material depends on the thermal conductivity and the thickness of that material. The thicker the insulation material, the less the thermal transmission [16]. In accordance with the scope of this work, 200 mm was selected as the maximum thickness for the analysis, representing the ordinary dimension employed in the current road cryogenic tanks [17]. The selection of an optimal thickness is related to obvious reasons of installation costs, size/weight of the tank, and thermal performance. Indeed, if the thickness increased too much, there would be more heat exchange surface, worsening the insulating capability of the system. The comparison between the insulating materials highlights that Cryogel ^®^ Z technology, due to the lower thermal conductivity than the perlite-based system, maintains a narrower Δ*T* in the LNG domain (from −20% for 5 mm thickness to −18% for 20 mm over perlite-based system), resulting in a better ability in insulate the cryogenic ambient. This effect is more evident for small thickness since, as the size of the insulating material increases, the contribution of the material’s heat transfer properties (i.e., thermal conductivity and thermal capacity) to its thermal resistivity competes with the thickness-effect of the insulator. The non-linear Δ*T* vs. *h* trend, which reflects the thermo-resistive behavior of the insulating materials as a function of their thickness, is in good agreement with that found by Hoseini et al. [18], who experimentally investigated the thermo-mechanical behavior of commercial aerogel blankets for insulating purposes. In agreement with the results of the FEM analysis, both insulating systems would maintain the temperature of the LNG after 96 h within its temperature equilibrium condition (−161 °C to −113 °C), therefore not risking total vaporization that may negatively affect the stability and safety of the LNG storage [19].

**Figure 3:**
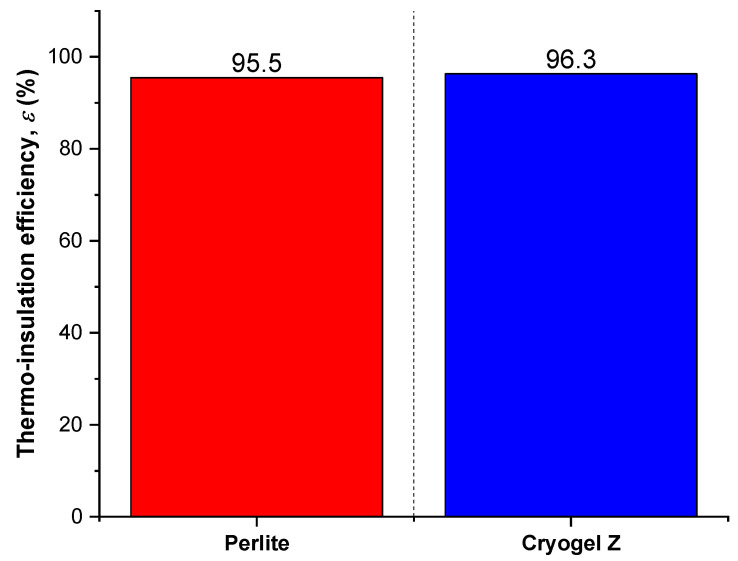
Thermo-insulation efficiency (*ε*) of perlite and Cryogel ^®^ Z systems at 96 h for 200 mm thickness.

The thermal performances of the two cryogenic insulation systems are more clearly summarized in Figure 3, where ε-values calculated at 96 h for 200 mm thickness are reported.

At the same thickness of insulation, Cryogel ^®^ Z allows for slightly lower heat losses than those obtained with insulation made with perlite + vacuum. Having ascertained an adequate thermal insulation efficiency for the aerogel technology, at this point it is worth analyzing what the implementation of this type of insulation setting would imply in terms of the weight of the cryogenic transport system. Consider the following data:

- Cavity volume of cryogenic tank for installing the insulator = 20.46 m^3^;
- Density of perlite = 60 kg/m^3^;
- Density of Cryogel ^®^ Z FRAB = 130 kg/m^3^.

In accordance with the data above, the required weights (*W*) of the two materials for creating the insulation system are:

$$W_{perlite}=20.46\;m^{3}\times 60\;\frac{\mathrm{kg}}{m^{3}}=1228\;\mathrm{kg}$$

$$W_{CryogelZ}=20.46\;m^{3}\times 130\;\frac{\mathrm{kg}}{m^{3}}=2660\;\mathrm{kg}$$

**Figure 4:**
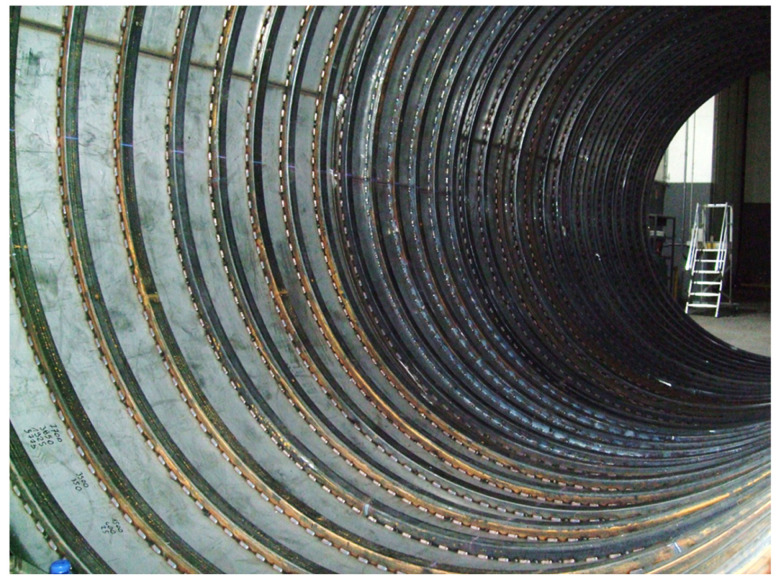
Internal structure of cryogenic tank detailing the circumferential stiffening rings used to prevent tank collapse due to vacuum conditions. The image has been kindly granted by A.L. CRYO srl company [21].

It would therefore seem that the use of Cryogel ^®^ Z increases the weight of the tank by about 116% with respect to the traditional perlite-based system. However, it must be considered that the insulation with perlite + vacuum makes it necessary to strengthen the external casing to be able to resist crushing due to the vacuum-preventing tank collapse phenomena. As illustrated in Figure 4, such an operation is carried out through circumferential stiffening rings welded on the internal surface of the tank casing [20,21]. Considering a center distance of about 220 mm, for a 13 m tank (analyzed in this work) about 13000/220 = 60 stiffening rings would be needed in adopting the perlite-based insulation system. The set of rings, generally made of S275JR structural steel, would contribute a total weight of 800 kg [17]. By using the Cryogel ^®^ Z system, the strengthening ring arrangement can be omitted, as it operates in the absence of vacuum condition, thus considerably reducing the weight increase compared to the perlite-based insulation by more than 50%. The above considerations were deduced by fixing the same thickness of the outer casing for the two types of insulating systems (perlite + vacuum and Cryogel ^®^ Z) for simplicity in the FEM analysis. However, in the case of the Cryogel ^®^ Z blanket, since it does not have to realize the vacuum condition, the airtight cavity is not necessary, and the outer casing has the sole purpose of protecting the insulating material. Therefore, other lightweight materials (e.g., aluminum) can also be used for the external casing replacing the steel, meaning the thickness can be reduced by at least 1 mm without any structural problems for the tank. These additional aspects make it possible to achieve, for the same volume of LNG transported, tanks insulated with Cryogel ^®^ Z with a lower tare weight than tanks insulated with the perlite + vacuum system.

### 2.2. Temperature Profile of LNG as a Function of the Storage/Transportation Time (t_st_)

**Figure 5:**
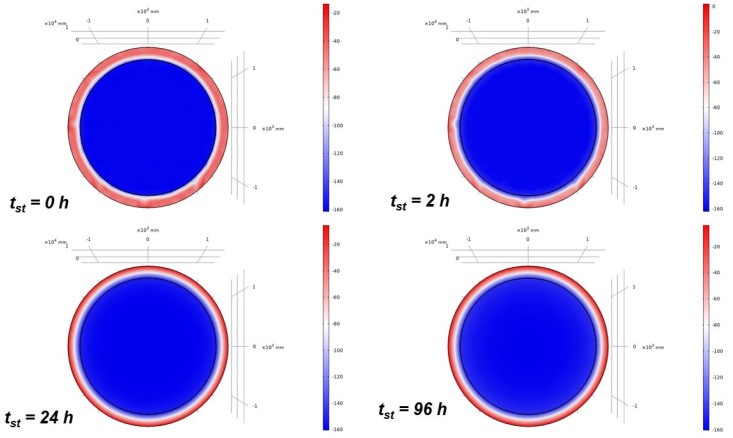
FEM analysis of the LNG temperature profiles obtained at *t_st_* = 0 h, 2 h, 24 h, and 96 h for the Cryogel-based insulating system.

This section reports the results of the time-dependent FEM analysis investigating the LNG temperature evolution as a function of *t_st_* by using perlite + vacuum and Cryogel ^®^ Z as insulating systems (200 mm thickness) in the cryogenic tank. Figure 5 graphically shows the temperature distribution results for different *t_st_* (0 h, 2 h, 24 h, and 96 h) to appreciate the thermal profile of LNG in response to the influence of the Cryogel-based insulating system.

**Figure 6:**
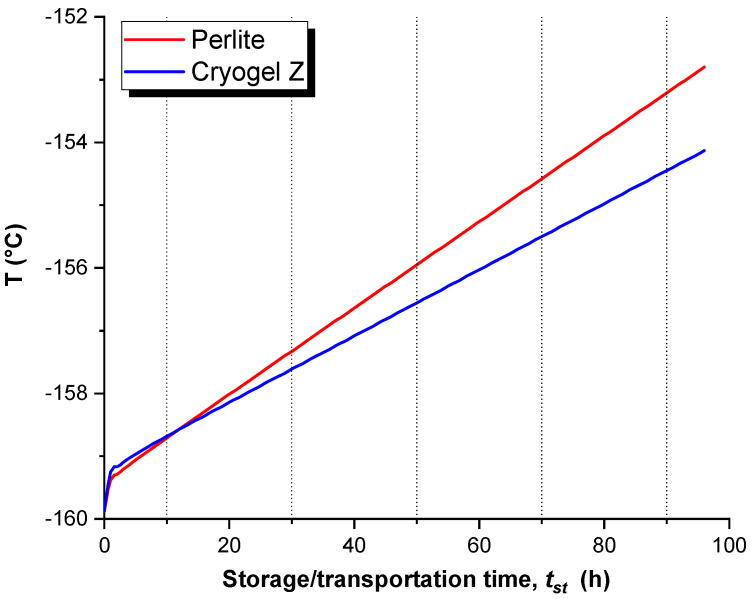
Temperature profiles of LNG as a function of *t_st_*.

Figure 6 plots the temperature variations as a function of *t_st_* for the two insulating systems under examination. At the beginning, the LNG temperature increases rapidly, due to the ingress of heat from outside. After about 10 h, different temperature trends are noted, exhibiting a linear increase but with different slopes. The slope reflects the ability of the insulating system to counteract the entry of heat from the outside, which causes a temperature increment of LNG. In support of the results obtained in the previous section, it is evident that the Cryogel ^®^ Z system, for the same *t_st_*, ensures a lower temperature increase rate than the perlite + vacuum system. The trend obtained by the FEM model is in good agreement with the results of previous numerical [22] and experimental analysis [23] addressed by other authors in the context of LNG storage in cryogenic conditions. Huerta and Vesovic [22] attributed the temperature increase with time to the growing ingress of heat from the outside towards the LNG domain because of an increase in the vapor area as the result of evaporation. Perez et al. [23] explained the early-time rapid change in temperature to the liquid–vapor transition of LNG, which is triggered once the initial liquefaction temperature of −160 °C is exceeded.

### 2.3. Boil-off Rate (BOR) Assessment

**Figure 7:**
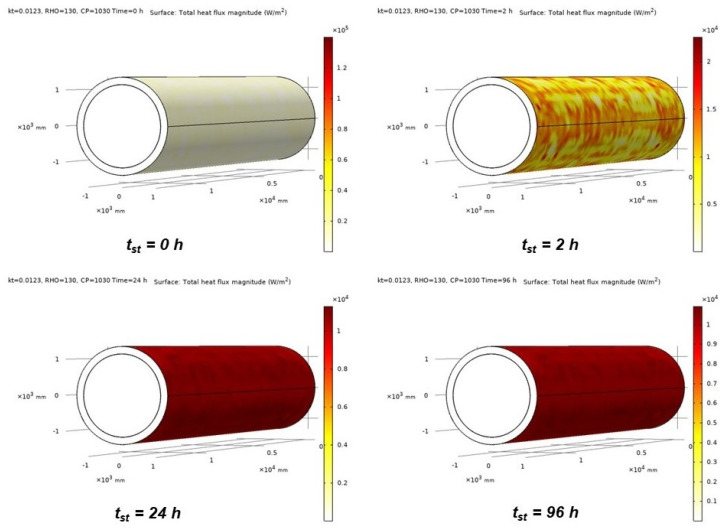
FEM analysis of the *Q* distribution obtained at *t_st_* = 0 h, 2 h, 24 h, and 96 h for the Cryogel-based insulating system.

*BOR* is recognized as one of the main indicators for evaluating the storage quality of cryogenic tanks; therefore, estimating its value is fundamental, especially in the selection of the constituent materials in the design phase. The ingress of heat into cargo tanks due to the difference between the inner temperature and the temperature of the external environment is the main reason for *BOR* generation [24]. Using the FEM model, the total heat flux (*Q*) on the external surface of tanks can be evaluated. Specifically, Figure 7 illustrates the *Q* distribution on the surface domain of the tank, insulated with the Cryogel ^®^ Z system (200 mm), at different *t_st_*, reflecting the heat exchange between the external ambient and the LNG domain over time. With the run of *t_st_*, the heat flux magnitude on the surface of the tank progressively increases, which therefore correlates with the increase in LNG temperature verified in Section 2.2.

**Figure 8:**
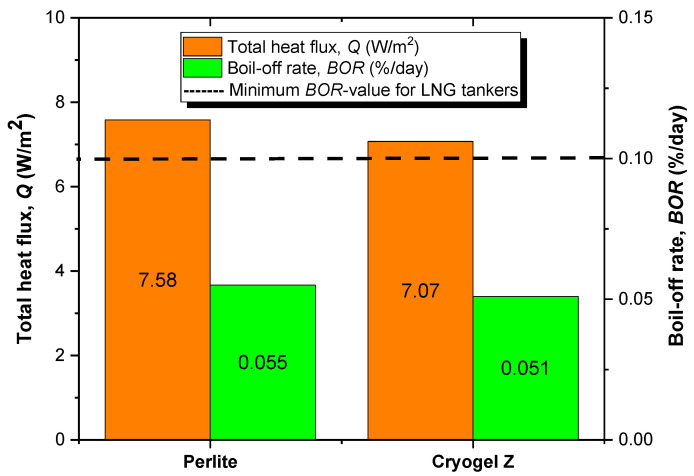
Total heat flux (*Q*) and boil-off rate (*BOR*) of perlite and Cryogel ^®^ Z systems at 96 h for 200 mm thickness.

Figure 8 plots the *Q*-values and *BORs* (at *t_st_* = 96 h) calculated according to the expression uniquely proposed in Refs. [1,24,25]. The Cryogel ^®^ Z insulator, ensuring a heat exchange about 7% lower than the perlite + vacuum counterpart, establishes lower *BOR*, thus demonstrating better thermal insulation performance in cryogenic storage. Furthermore, the data obtained from FEM simulation would underestimate the actual potentiality of FRAB technology for the examined application. Indeed, the possibility of working in ambient pressure conditions (no vacuum) makes it possible to gain space in the tank for LNG storage. This has two interesting implications: 1) with the same tank capacity, it is possible to achieve a greater LNG transport/storage yield without excessive weight increases; 2) the possibility of transporting a greater volumetric quantity of LNG would more effectively counteract heat leakage phenomena in the tank, furtherly reducing the *BOR*. The latter evidence is well supported by the research of Khan et al. [26]: the greater the filling of the tank, the lower the vapor area in the liquid–vapor interface for heat transfer and therefore the lower the heat losses to which LNG will be subjected during its storage/transportation. At this rate of heat transfer and voyage time, the *BOR* from the tank for both insulating systems is below the typical minimal value (0.10%/day) for LNG tankers in laden voyage conditions outlined in the International Group of Liquefied Natural Gas Importers report [24]. Although this result would seem promising in terms of the thermo-insulating efficiency of the implemented insulating systems, it is necessary to consider some approximations made in the construction of the computational model, which would neglect potential sources of thermal losses of the tank in real conditions:

- For simplicity, the tank was modeled as an axially symmetrical cylinder where only the heat exchanges between the shell and the external ambient were examined, therefore neglecting the presence and the thermal effect of the semi-elliptic endings that commonly characterize real designs.
- The model neglects the presence of connections between the various components of the tank (filling connections, valves, pressure gauges).
- The simulation does not consider the influence of isolated supports between the external and internal cladding of the tank.
- The anchor supports comonly used to secure the tank to the truck were omitted in the numerical analysis. Even if they are external to the thermal insulating layer, the anchor supports must be carefully analyzed and designed to minimize the thermal bridges between the external shell and the vehicle.

The impact of these contributions on the efficiency of LNG cryogenic systems will be the main topic in the next studies for refining the proposed FEM model, by providing information closer to real working conditions of tanks that will be useful for design purposes.

### 2.4. Estimating the Impact of Sloshing Conditions on BOR

In the previous section, *BOR* results were obtained by implementing an FEM model considering static storage conditions by neglecting the dynamic motions of the LNG inside the tank. In the real situation, during LNG transportation, whether using carriers for marine transportation or road tankers for land transportation, the sloshing and mechanical vibration of the tank are inevitable, aggravating the boil-off gas generation [27]. Under static conditions, the liquid–vapor interface approaches equilibrium by forming a temperature stratification layer below and above the LNG surface, accompanied by mild pressure variations. When a tank experiences sloshing, there are two reasons for boil-off gas generation. Firstly, the existence of sloshing excitation intensifies the heat and mass transfer between the liquid and vapor phases, which increases the internal energy of LNG, promoting its evaporation. Secondly, due to the existence of viscosity, the mechanical energy of the tank sloshing is transformed into the internal energy of LNG, resulting in the increment of LNG temperature, thus causing gas evaporation [23,27]. Although estimating the impact of sloshing provides a more realistic assessment of the insulating performance of a cryogenic tank, its prediction appears decidedly complex considering that the phenomenon is non-linear and stochastic in nature. Moreover, there are just a few well-targeted computational fluid dynamics (CFD) simulating software programs that have been implemented for the study of vibratory phenomena in cryogenic tanks, including Ansys Fluent, Aspen Plus, and Flow 3D. General multi-physics interfaces, such as COMSOL, require huge and unsustainable computational efforts to obtain accurate and high-fidelity results. Considering the non-viability of implementing the sloshing effect in the developed FEM model, its contribution was estimated from the results obtained in previous research works by adapting them to the tank design model of this study.

First, it was extensively demonstrated that heat losses due to sloshing occur significantly at a specific frequency, namely the natural frequency of the liquid column (*f_n_*), where the maximum resonance condition occurs [28]. If the shape of the tank is known, *f_n_* is determined by the depth of LNG and the shape parameters of the tank as follows (Equation (1)):

(1) $$f_{n}=\frac{1}{2\pi }\sqrt{\frac{\pi \cdot g\cdot \tanh\left(\frac{\pi \cdot d}{l}\right)}{l}}$$

where *d* is the fluid depth (in m), *l* is the length of the cylinder (in m), and *g* is the gravity acceleration (9.81 m/s^2^). Using Equation (1), *f_n_* of the LNG tank in this paper is about 0.77 Hz under the conditions *l* = 1.3 m and *d* = 1.1415 m (100% filling ratio condition).

**Figure 9:**
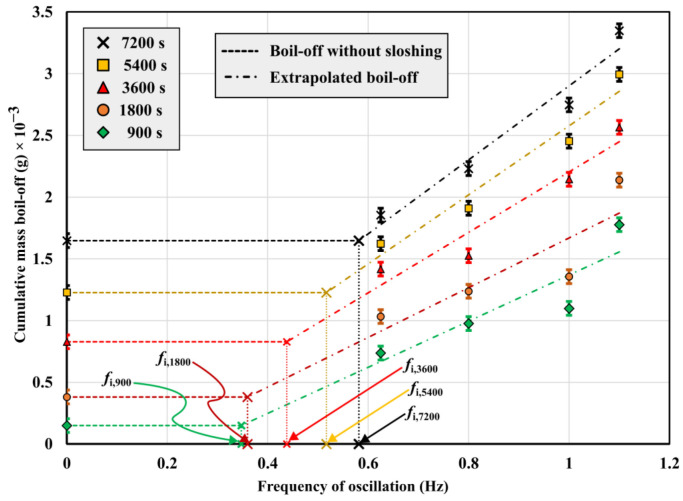
Boil-off gas generation vs. frequency of oscillation. Reproduced with permission from [29].

By referring to the results of the analysis from Nitin et al. [29], who conducted a comprehensive investigation into the relationship between sloshing frequency and boil-off gas generation in mobile cryogenic vessels, the *BOR* growth rate at 0.77 Hz was estimated. The plot from Ref. [29] in Figure 9 shows that, at this frequency, the increase in the boil-off gas generation is about 44% compared to the static storage conditions. This contribution can then be added to the *BOR* values mentioned in Section 2.3, resulting in 0.079%/day and 0.073%/day for perlite and Cryogel ^®^ Z systems, respectively.

**Figure 10:**
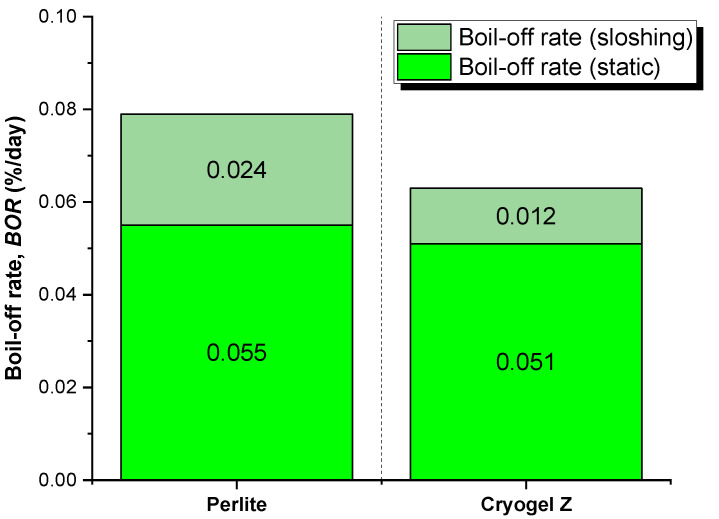
Sloshing contribution in boil-off rate (*BOR*) for perlite and Cryogel ^®^ Z systems at 96 h.

In this calculation, however, the influence of damping from the insulating layer was not considered. The damping factor of the materials used, like cryogenic insulators, plays a key role in suppressing the sloshing and therefore in preventing the generation of boil-off gas. To assess the influence of this contribution, the research work from Fesmire et al. [30], who studied the thermo-mechanical performance of different insulation systems in cryogenic tanks under vibration cycles, was considered. The results highlighted that, under non-vacuum conditions, the aerogel technology insulator provided a heat leak under a frequency regime about 50% lower than perlite-based systems. Aerogel showed advantages over perlite in higher damping and minimal mechanical settling. Therefore, in consideration of this contribution, the overall *BOR* of the Cryogel ^®^ Z-based tank will be equal to 0.063, which is about 20% lower than those of the perlite insulating system (Figure 10).

### 2.5. Thermo-Economic Assessment

To gain insight into the economic view of the problem, *BOR* values estimated in the previous section were implemented in a calculation model to compare perlite + vacuum and Cryogel ^®^ Z systems in terms of material and money savings. The cryogenic tank was modeled with a storage volume of 50 m^3^. By considering the LNG density of 468.1 kg/m^3^, a totally filled tank contains 23,405 kg of LNG. For the perlite-based insulated tank, *BOR* of 0.079%/day corresponds to approximately 18.48 kg of LNG evaporating per day. Implementing the Cryogel ^®^ Z insulating system, an evaporation rate of 14.75 kg of LNG would occur, resulting in savings of approximately 16 kg of LNG for a 96-h voyage. For an LNG cost of 0.36 EUR/kg, 6.65 EUR are expected to be lost per voyage. On the other hand, FRAB technology would decrease economic losses by about 20%, resulting in a loss of 5.31 EUR per 96-h voyage.

## 3. Conclusions

Thermal insulation plays a crucial role in the design and construction of cryogenic tanks. The storage effectiveness of these systems strictly depends on applied materials in the insulating layer. The present work proposed an FEM-based study of the possibility of implementing FRAB technology in the design of cryogenic tanks for the storage and transport of LNG, intending to achieve adequate heat insulation performance without the need to realize vacuum arrangements (typically necessary for traditional bulk-fill and MLI systems). The results of the numerical analysis showed that the FRAB Cryogel ^®^ Z system would be a potential candidate to replace perlite vacuum-based insulators. The Cryogel-insulated tank highlighted a higher specific thermo-insulating efficiency (+0.9%) and a *BOR* value approximately 7% (in static condition) and 20% (estimating sloshing effect) lower than perlite-based insulation, reflecting the typical performance found in LNG tankers under real voyage conditions (*t_st_* of 96 h and outside temperature from the environment of 50 °C). Additionally, FEM analysis demonstrated that the thermal insulation effect is enhanced with the increase of the insulator thickness. At the same thickness, Cryogel ^®^ Z ensured a lower temperature gradient inside the LNG domain than the perlite + vacuum configuration (from −20% for 5 mm thickness to −18% for 20 mm), demonstrating better capacity to avoid heat leakage during LNG transportation. Ultimately, implementing aerogel technology would benefit several aspects, including reduced weight, simplified design, safety, cost savings, and sustainability. The next steps of the research activity will be focused primarily on strengthening the predictive FEM model. Many industrial application fields, such as loading/unloading problems with cryogenic liquids accompanying the time change, heat loss problems of local support structures, and dynamics problems with sloshing during operation, will be solved by this type of numerical analysis. The high-fidelity calculation method will be greatly beneficial to the analysis, design, and development of the LNG tank system. The validation of the computational results will therefore require a scale-up of the FRAB system on pilot systems first and then on real tankers.

## 4. Materials and Methods

### 4.1. Materials

#### 4.1.1. Cryogel ^®^ Z

**Figure 11:**
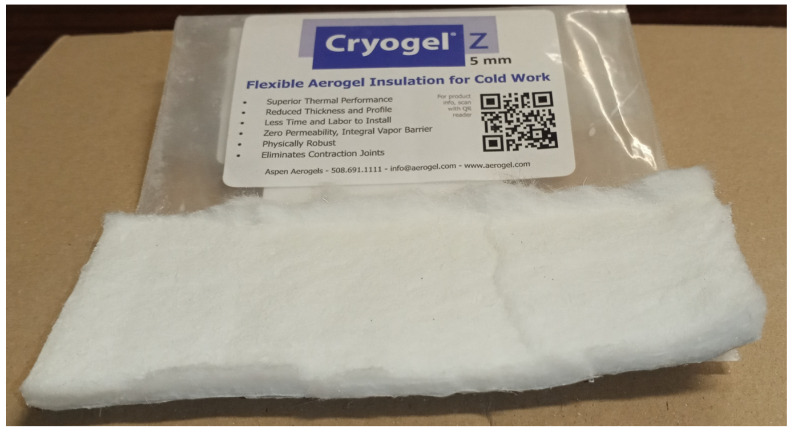
Cryogel ^®^ Z sample.

Cryogel ^®^ Z, produced by Aspen Aerogels Inc. (Northborough, MA, USA), was selected in this study to assess the feasibility of implementing FRAB technology in the thermal insulation of LNG cryogenic tanks. A blanket sample, kindly provided by Teknowool Roma SRL (Rome, Italy) as part of this research work, is shown in Figure 11. The sample looks like a flexible aerogel blanket with a factory-applied vapor barrier intended for sub-ambient and cryogenic applications.

**Figure 12:**
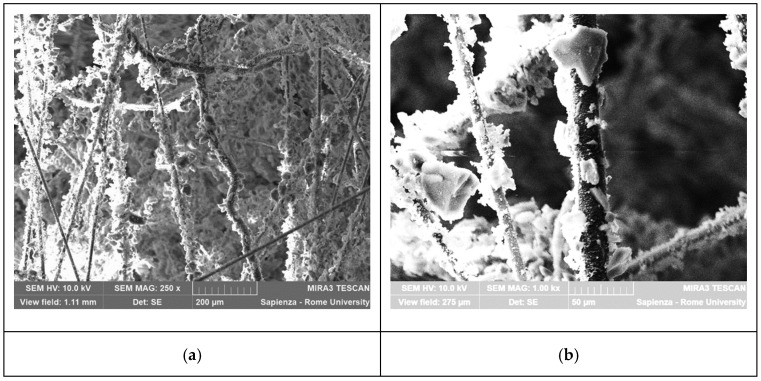
SEM micrographs of Cryogel ^®^ Z at different magnifications: (**a**) 250× and (**b**) 1000×.

The test sample was first analyzed by scanning electron microscopy (SEM) to investigate its microstructure. SEM was conducted by using a Tescan MIRA 3 SEM (Tescan, Brno, Czech Republic) on a square specimen of 1.5 cm per side previously graphitized to make it conductive for microscopy analysis. A graphite sputter coater (EM SCD005, Leica, Wetzlar, Germany) was used for the sample preparation. Figure 12a,b shows the SEM micrographs of the Cryogel ^®^ Z acquired in secondary electron (SE) emission mode at different magnifications.

From the SEM analysis, the glass and PET fibers coated with amorphous silica aerogel particles can be observed. The fibrous network shows voids that contribute to the thermal insulation performance of the material. In accordance with the manufacturer’s declaration, there are no fiber–fiber contacts within the aerogel–fiber composite matrix that would allow solid heat conduction through aerogels [8]. The physical properties of the Cryogel ^®^ Z, for the FEM model, were taken from the technical datasheet of the material as listed in Table 1.

#### 4.1.2. Perlite + Vacuum

The information and data for the comparison insulating system “perlite + vacuum” was taken from Beikircher and Rottmann’s research work [31], reporting an experimental study on evacuated perlite powders for super-insulated long-term storage applications. The material’s parameters implemented in the FEM simulation are listed in Table 2.

### 4.2. FEM Modeling Method

#### 4.2.1. Aim of FEM Analysis

In this work, the possibility of implementing FRAB technology in the design of cryogenic tanks for LNG storage was investigated through numerical analysis. The COMSOL Multiphysics simulation has made it possible to obtain a predictive analysis of the behavior of these insulating materials in comparison with the systems currently commonly used in the application under examination. The purpose of the simulation was to study the variation over time of the average temperature in the LNG volume for 96-h storage under specific boundary conditions, by evaluating which of the insulation systems studied has the best heat insulation performance in terms of heat exchange, ε, and *BOR*.

#### 4.2.2. Model Geometry and Materials

**Figure 13:**
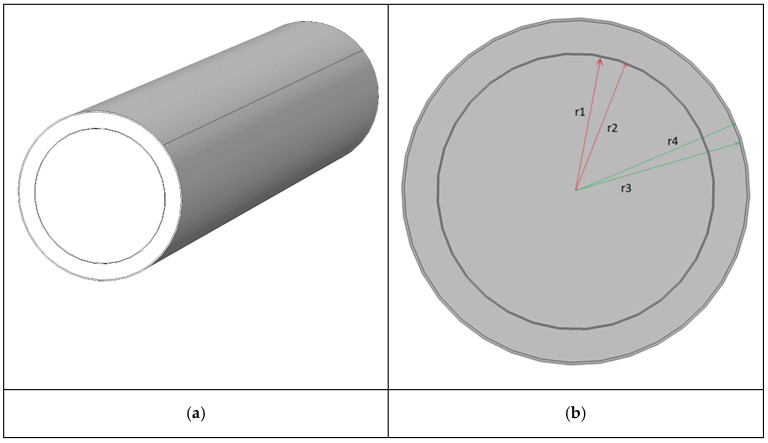
FEM model geometry: (**a**) 3D model and (**b**) cross-section detailing the inner domains of the tank.

In the simulation, a simplified geometry was chosen, consisting of a single cylindrical body (Figure 13a) that represents the lateral surface of the cryogenic tank. As mentioned in Section 2.3, the geometry used in the model is subject to simplifications as the tank ends, bearings, and connections with the truck have been omitted, focusing on the heat exchange along the lateral surface. The model geometry involved four concentric cylinders 1.3 m in length. The cross-section in Figure 13b highlights the four domains constituting the internal arrangement of the tank:

- Domain 1 (0 ➔ r_1_ = 1.1415 m). Volume of the cryogenic tank containing LNG. For the physical properties of the material (density, thermal conductivity, and heat capacity at constant pressure), the data reported in the COMSOL Multiphysics library, identified as *CH4 (methane) [liquid]*, were used.
- Domain 2 (r_1_ ➔ r_2_ = 1.1475 m). Thickness of the internal metal shell of the cryogenic tank. A chromium stainless steel from the COMSOL Multiphysics library was used as a material for this domain, identified as *Stainless Steel Chromium Steel*.
- Domain 3 (r_2_ ➔ r_3_ = 50 mm—100 mm—150 mm—200 mm). Domain corresponding to the insulation system of the cryogenic tank. Two heat-insulating solutions (Cryogel ^®^ Z and perlite + vacuum) and the influence of the thickness layer were assessed. Materials’ parameters are reported above in Table 1 and Table 2, respectively.
- Domain 4 (r_3_ ➔ r_4_ = 2.5 mm). Thickness of the external metal jacket of the cryogenic tank. The same material implemented for Domain 2 was used.

The dimensions of the tank model design complied with the technical specifications kindly provided by AE PROJECT srl (Rome, Italy), a multidisciplinary architectural and engineering company operating in the design of plants for the storage and distribution of LNG [17].

#### 4.2.3. Physics and Boundary Conditions

A time-dependent numerical study was performed in COMSOL Multiphysics to simulate the conduction and convection heat transfer in LNG as a function of the insulating system. The “Heat Transfer in Solids and Fluids” physics interface was used for this purpose.

For the solid domains (Domains 2, 3, and 4), the heat transfer is dominated by conduction. The governing equations are Equations (2) and (3):

(2) $${\rho C}_{p}\frac{\delta T}{\delta t}+{\rho C}_{p}u\cdot \nabla T+\nabla \cdot q=Q+Q_{ted}$$

(3) q=−k ∇T

where *C_p_* is the heat capacity (in J/kg × K), *ρ* is the fluid density (in kg/m^3^), *u* is the flow velocity (in m/s), *T* is the temperature (in K), *q* is the heat flux (W/m^2^), *Q* is the heat source (in J), *Q_ted_* is the work due to thermoelastic damping (in J), and *k* is the thermal conductivity (in W/m × K).

For the LNG domain (Domain 1), the heat transfer mechanism from the thermal fluid to the wall of the cryogenic tank considers the thermal contribution due to the change in pressure (denoted as *Q_p_*) and the heat generated by viscous dissipation of the fluid (denoted as *Q_vd_*). The energy equation, coupled to Equation (3) for heat conduction, that needs to be solved in that case is given by Equation (4):

(4) $$\rho C_{p}\frac{\delta T}{\delta t}+\rho C_{p}u\cdot \nabla T+\nabla \cdot q=Q+Q_{p}+Q_{vd}$$

Table 3 presents the values used for the initial boundary conditions to solve the numerical problem. The computation performed using these boundary conditions provides conservative results. Domain 4 is initially set to 50 °C temperature, reflecting the maximum environment temperature that can be faced in Europe during the LNG transport in summer. This setting would assume that the external jacket of the tanker is in thermal equilibrium with the outside temperature from the environment. The initial temperature and pressure of LNG were set to −160 °C and 5 atm respectively, which are typical loading conditions of cryogenic tanks. The temperature of the insulating layer (−50 °C) is an average value between the initial temperature of the LNG (Domain 1) and the temperature of the external metal jacket of the tank (Domain 4). Although in real applications the perlite-based insulating system provides the coexistence of a “material” phase and the “vacuum” phase, for simplicity in this simulation, the insulating layer was modeled as a mono-material domain. Indeed, the material’s properties in Table 2 already refer to the physical and thermal characteristics of perlite in vacuum conditions for cryogenic applications. Starting from such an initial setting, the time-dependent analysis is expected to determine the heat transfer mechanisms and the thermal profile of LNG in a simulation time of 96 h, assuming a journey of the cryogenic tanker of 1000 km, which considers possible impediments during the travel (road queues, slowdowns, stops for the driver to rest, road blocks due to events, breakages, punctures of the tires, etc.).

#### 4.2.4. Meshing

In the model under consideration, a physics-controlled mesh with fine-sized triangular elements was chosen. A mesh convergence study was performed, and a maximum element size of 104 mm was selected to ensure the accuracy of the numerical results.

#### 4.2.5. Output Results

The prediction of the LNG temperature profile both as a function of *h* and *t_st_* as well as the assessment of the *Q* distribution over *t_st_* on the cryogenic tank model led to the determination of two performance indicators aimed at determining the thermal insulation effectiveness of the two insulating systems (Cryogel ^®^ Z and perlite + vacuum): *ε* and *BOR*.

After determining the temperature profile of LNG over the time range of 96 h, the *ε*-index was defined as follows (Equation (5)):

(5) $$\varepsilon =\frac{T_{f}}{T_{0}}\times 100\;\left(\%\right)$$

where *T_f_* is the temperature reached by the LNG domain after 96 h, and *T_0_* is the initial temperature of LNG (−160 °C). This indicator provides a measure of the cryogenic system’s performance in maintaining proper LNG temperature throughout storage and transportation.

*BOR* is estimated by using Equation (6) in accordance with the well-recognized expression used in the framework design of the LNG cryogenic system [1,24,25]:

(6) $$BOR=\frac{Q\times 86,400}{\Delta H\times V\times \rho_{\mathrm{LNG}}}\;\left(\%/day\right)$$

where *Q* represents the total heat flux that penetrates the cryogenic tank, Δ*H* is the vaporization latent heat of LNG (5.1 × 10^5^ J/kg), *V* is the cargo capacity (50 m^3^), and *ρ*_LNG_ is the LNG density (468.1 kg/m^3^). This indicator represents an estimation of the quantity of evaporated cargo presented as a loss of the total volume of liquid cargo during a single day.

## Figures and Tables

**Table 1:** Physical properties of Cryogel ^®^ Z implemented in FEM analysis.

Property	Value
Density	130 kg/m^3^
Thermal conductivity in cryogenic conditions (<−100 °C)	0.012 W/m × K
Heat capacity	1030 J/kg × K

**Table 2:** Physical properties of perlite + vacuum system implemented in FEM analysis.

Property	Value
Density	60 kg/m^3^
Thermal conductivity in cryogenic conditions (<−100 °C)	0.016 W/m × K
Heat capacity	387 J/kg × K

**Table 3:** Boundary conditions implemented in FEM analysis.

Boundary Condition	Value
Initial temperature of LNG (Domain 1)	−160 °C
Initial pressure of LNG (Domain 1)	5 atm
Initial temperature of the internal metal shell (Domain 2)	−160 °C
Initial temperature of the insulating system (Domain 3)	−50 °C
Initial temperature of the of the external metal jacket (Domain 4)	50 °C

## Data Availability

The data presented in this study, supporting the results, are available in the main text. Additional data are available upon request from the corresponding authors.
